# *Trypanosoma vivax* Infections: Pushing Ahead with Mouse Models for the Study of *Nagana*. I. Parasitological, Hematological and Pathological Parameters

**DOI:** 10.1371/journal.pntd.0000792

**Published:** 2010-08-10

**Authors:** Nathalie Chamond, Alain Cosson, Marie Christine Blom-Potar, Grégory Jouvion, Simon D'Archivio, Mathieu Medina, Sabrina Droin-Bergère, Michel Huerre, Sophie Goyard, Paola Minoprio

**Affiliations:** 1 Laboratoire d'Immunobiologie des Infections à Trypanosoma, Département d'Immunologie, Institut Pasteur, Paris, France; 2 Unité de Recherche et d'Expertise Histotechnologie et Pathologie, Institut Pasteur, Paris, France; New York University School of Medicine, United States of America

## Abstract

African trypanosomiasis is a severe parasitic disease that affects both humans and livestock. Several different species may cause animal trypanosomosis and although *Trypanosoma vivax* (sub-genus *Duttonella*) is currently responsible for the vast majority of debilitating cases causing great economic hardship in West Africa and South America, little is known about its biology and interaction with its hosts. Relatively speaking, *T. vivax* has been more than neglected despite an urgent need to develop efficient control strategies. Some pioneering rodent models were developed to circumvent the difficulties of working with livestock, but disappointedly were for the most part discontinued decades ago. To gain more insight into the biology of *T. vivax*, its interactions with the host and consequently its pathogenesis, we have developed a number of reproducible murine models using a parasite isolate that is infectious for rodents. Firstly, we analyzed the parasitical characteristics of the infection using inbred and outbred mouse strains to compare the impact of host genetic background on the infection and on survival rates. Hematological studies showed that the infection gave rise to severe anemia, and histopathological investigations in various organs showed multifocal inflammatory infiltrates associated with extramedullary hematopoiesis in the liver, and cerebral edema. The models developed are consistent with field observations and pave the way for subsequent in-depth studies into the pathogenesis of *T. vivax* - trypanosomosis.

## Author Summary

While most research efforts have focused on *T. b. brucei* trypanosomosis, infections caused by *T. vivax* and *T. congolense* which predominate in livestock and small ruminants have been subject to little study. In order to circumvent the major constraints inherent to studying *T. vivax*/host interactions in the field, we developed *in vivo* murine models of *T. vivax* trypanosomosis. We show here that the mouse experimental model reproduce most features of the infection in cattle. More than reflecting only the main parasitological parameters of the animal infection, the mouse model can be used to elucidate the immunopathological mechanisms involved in parasite evasion and persistence, and the tissue damage seen during infection and disease. Studies planned for the future will allow us to further investigate *T. vivax*–induced immunopathology in an experimental context for which all the necessary tools are now available.

## Introduction

African trypanosomiasis, one of the most neglected diseases, consists of a number of important human and animal pathologies caused by parasitic protists of the order Kinetoplastida. Human African Trypanosomiasis (HAT), or sleeping sickness, and animal trypanosomosis, or *Nagana*, are vector-borne diseases, that are primarily cyclically transmitted by tsetse flies. HAT is a major public health problem in 35 sub-Saharan countries. The related animal challenge, caused by several species, i.e. *Trypanosoma vivax*, *Trypanosoma congolense* and to a lesser extent to *Trypanosoma brucei brucei* causes about 3 million deaths annually in cattle and has a marked impact on African agriculture, causing annual livestock production losses of about US$ 1.2 billion. *T. vivax* accounts for up to half of total *Trypanosoma* prevalence in West Africa where it is considered the major pathogen for livestock and small ruminants [1],[2],[3]. Outside tsetse endemic areas, West African *T. vivax* isolates were introduced long ago into South American countries where it represents a real threat since it can be efficiently transmitted across vertebrate hosts by mechanical means and by various biting flies and tabanids [4], [5], [6].

The severity of the disease depends on parasite strain, endemicity and host species, but the key steps in the *T. vivax* - host interactions are still largely unknown. Several pieces of evidence point to the importance of host genetic factors in determining individual susceptibility and/or resistance to this infection [3], [7], [8], [9], [10], [11]. *Trypanotolerance* is defined as the ability demonstrated by cattle of different genetic backgrounds to control trypanosomosis [12], [13]. It has previously been reported that increased bovine resistance to trypanosomosis is associated with more control over parasitemia and related anemia, two of the main pathogenic effects of trypanosome infections [14], [15]. However, dissimilar courses of the infection may be due to genetic polymorphism and to the virulence of the parasite isolates, thus leading to moderate, progressive and/or lethal pathologies and therefore affecting mortality rates [5], [6], [7].

It is widely accepted that if trypanosomosis is to be successfully treated in the field, a number of parameters must be taken into account, including the seasonal trypanosome prevalence and vector abundance, the severity of the disease, the magnitude of the anemia, the stock nutritional state and the prescription of an appropriate trypanocidal drug [6], [16], [17], [18]. However, the antigenic complexity of trypanosomes, their ability to expose a variety of genetically-controlled surface coat antigens (VSG), and the diversity of the immune responses presented by unrelated hosts [19], [20], [21], call for the discovery of new parasite genetic markers and more in-depth knowledge of host trypanotolerance mechanisms.

Several early studies were conducted in more affordable mouse or rat experimental models of infection in attempts to throw light on trypanotolerance, antigenic variation, the pathogenesis of intravascular coagulation, and *T. vivax* immunobiology and dynamics [7], [11], [19], [22], [23], [24], [25]. However, these studies used a variety of more or less virulent isolates from cattle, goats, sheep, horses and donkeys to explore the ability of *T. vivax* stocks to infect several intact or immunosuppressed mouse strains. Although these studies had a huge impact on research into *T. vivax*, the diversity of the results they yielded and the difficulties encountered in establishing axenic parasite cultures or reliable *in vivo* infections that entirely resemble natural infections [26], constrained the work performed with these models. In consequence, more than 20 years ago, while biological investigations into VSG and the identification of serodemas were usual for more than a few trypanosomes of the *Trypanozoon subgenus, studies on T. (Dutonella) vivax VSG molecules and structure of the coat were just been encouraged [26]. Research into T. vivax then focused on characterizing parasite surface proteins or comparing genetic diversity of Western to Eastern African parasite stocks and more recently on analyzing population clonality, [10], [27], [28], [29], [30], [31], [32], [33], but somehow neglected the further development of suitable rodent models.*

Now, and in an attempt to circumvent the major constraints inherent to studying *T. vivax*/host interactions in the field and data inconsistencies arising from the difficulties encountered in the past, we have developed *in vivo* murine models of trypanosomosis using a *T. vivax* isolate known to maintain infectivity to rodents [23]. Here we show that this *T. vivax* isolate retains its original characteristics after several years of cryopreservation. The parasites can grow, multiply and be transmitted *in vivo* following predictable kinetics in the peripheral blood of different mouse strains selected for their susceptibility or resistance to different parasite inocula. Sustained and reproducible infections are obtained that successfully mimic the dynamics of the parasitological, histological and pathological features of the infection and closely resembling those observed for cattle trypanosomosis in the field. We have thus developed reliable mouse research models that can be used to elucidate the immunopathological mechanisms involved in *T. vivax* infection and associated disease. It is worth noting that *T. vivax* was recently shown to express a functional gene involved in the non specific polyclonal activation of host B cells and that this gene is absent in more widely studied *T. brucei* and *T. congolense* [34]. Furthermore, the work presented here is expected to be a useful and complementary tool for the further studies of *T. vivax* immunobiology and will thus provide valuable information about trypanotolerance, *Trypanosoma* evasion strategies from host immune system, and immunopathogenesis.

## Materials and Methods

### *T. vivax* parasites and oligonucleotides

*Trypanosoma (Dutonella) vivax* stabilates (STIB 731-A), cryopreserved on September 25, 1996 after 9 passages in mice, were kindly provided by R. Brun (Swiss Tropical Institute, Basel, Switzerland). STIB 731-A stabilates were originally prepared in November 1982 using bloodstream forms of IL 1392 *T. vivax* stock obtained from the blood of goat #M918, at ILRAD (ILRI), Nairobi, Kenya. This West African IL 1392 goat stock was derived from the Zaria Y486 Nigerian isolate of a naturally infected Zebu steer maintained by 62 serial passages in mice [23]. VSG ILDat 1.2 (ILRAD Duttonella antigen type 1.2) specific primers were deduced from the VSG ILDat 1.2 full length sequence (TvY486_0004810 variant surface glycoprotein putative, 1215 bp) obtained from the GeneDB of the Zaria Y486 *Trypanosoma vivax* nuclear genome (Sanger Institute Pathogen Sequencing Unit (PSU), http://www.sanger.ac.uk/Projects/T_vivax/): VSG-1.2F (5′ AATTTTGGTGAGTGTCGGTGT 3') and VSG-1.2R (5' ATTTCCTCCACCACGTAGCTC 3'). *T. vivax*- specific forward and reverse ribosomal promoter primers were also deduced from the Zaria Y486 chromosome 3: TvrDNAF (5' CTGATTTCGCCACTGCTATTATTTGC 3') and TvrDNAR (5' CGCTTCACTTGATGATCGTTTCG 3'), respectively. Parasites were maintained by weekly passages in mice and new stabilates were appropriately and regularly frozen in polysoma buffer/glycerol, as previously described [11]. Blood smears were prepared from infected mouse blood, air dried, fixed in methanol for 5 minutes and further stained with 5% Giemsa for 20 minutes.

### Mice and infections

Seven to 10-week-old male BALB/c (H2^d^), C57BL/6 (H2^b^) or Swiss outbred (CD-1, RJOrl:SWISS) mice (Janvier, France) were used in all the studies. Mice were injected intraperitoneally with 10^1^–10^5^ bloodstream forms of *T. vivax* obtained at the peak of parasitemia (day 8 post infection). For parasite enumeration, five microliters of blood were harvested individually from the tail vein and appropriately diluted in buffered saline when necessary. Blood parasite counts were established under a light microscope and expressed as number of parasites per milliliter of blood. All animal work was conducted in accordance with relevant national and international guidelines (see here below).

### Ethics statement

All mice were housed in our animal care facilities in compliance with European animal welfare regulations. The Institut Pasteur is member of the Committee #1 of the Comité Régional d'Ethique pour l'Expérimentation Animale (CREEA), Ile de France. The Animal housing conditions and protocols used in the present work were previously approved by the “Direction des Transports et de la Protection du Public, Sous-Direction de la Protection Sanitaire et de l'Environnement, Police Sanitaire des Animaux” under the number B 75-15-28 accordingly to the Ethics Chart of animal experimentation which includes appropriate procedures to minimize pain and animal suffering. PM has permission to perform experiments on vertebrate animals #75-846 issued by the Department of Veterinary Services of Paris, DDSV and is responsible for all the experiments and protocols carried out personally or under her direction in the framework of laws and regulations relating to the protection of animals.

### Hematology

50 µl of retro-orbital blood were recovered onto 0.5 M EDTA. Samples were analyzed in a Scil Vet abc (Scil, Strasbourg, France) using pre-established and normalized parameters for the different mouse strains. Peripheral reticulocytes were counted as described [35], modified by S. Bagot (personal communication). Briefly, 5 µl of blood were fixed in 1 ml of 0.25% glutaraldehyde in PBS pH 7.4 and further stained with 1 µM Hoechst 33258/Thiazole orange 0.1 µg/ml in PBS pH 7.4 for 1 h at 37°C.

### Histopathology

Twenty days after infection, mice were anesthetized with an i.p. injection of 0.1 ml per 10 g mouse body weight of a solution containing 1 mg/ml xylazine (Rompun 2%, Bayer, Leverkusen, Germany) and 10 mg/ml ketamine (Imalgène 1000, Merial, Lyon, France), and then sacrificed by cervical dislocation. After a complete post-mortem examination, the spleen, liver, kidneys, lung, heart and specimens of the central nervous system were removed and immediately fixed in 10% neutral-buffered formalin. Tissue samples from these organs were embedded in paraffin; five-micrometer sections were cut and stained with hematoxylin and eosin (HE).

### Statistical analyses

All the experiments were performed two or three times using at least 5 mice per experimental group and per time point. Mice were analyzed individually and the differences between the groups used in this study were tested for statistical significance using Student's test or the Log-rank (Mantel-Cox) test whenever appropriate (Prism software, Graph Pad, San Diego, CA). Data are expressed as arithmetic means are presented as arithmetic means +/− the standard deviation (SD) of the means.

## Results

### *Trypanosoma vivax* molecular and phenotypic characterization

**Figure 1:**
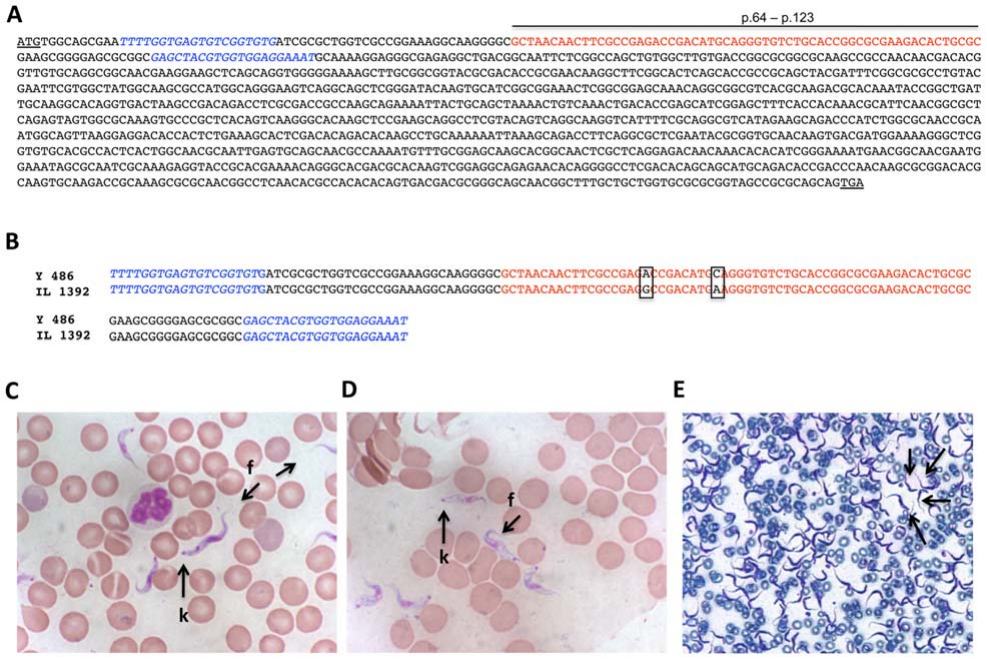
Molecular identity of *T. vivax* IL 1392. Full lengh of *ILDat1.2 VSG* gene (A). Initiation and stop codons are underlined. The specific *T. vivax* 20-amino acids sequence at the N-terminal end of the gene is depicted in red (positions 64 to 123). Forward and reverse primers used in this experiment are in italics. DNA was extracted from *T. vivax* bloodstream forms and amplified by PCR using *VSG-1.2*F and *VSG-1.2*R primers. A fragment of 148 bp was obtained and the resulting sequence aligned with the Y486 reference strain (B). Two point mutations are squared. Blood smears of a mouse infected with *T. vivax* were fixed and stained with Giemsa (C and D); k = kinetoplast, f = flagellum. The high number of circulating parasites at the peak of parasitemia can be evaluated in the picture (E).

The IL 1392 West African stock of *T. vivax* is derived from the Nigerian isolate Zaria Y486 [23] which is infective for rodents and can be cyclically and/or mechanically transmitted [36], [37]. Rodent-infective derived clones of Y486 *T. vivax*, notably the IL 1392, have already been shown to express VSG ILDat 1.2 (ILRAD *Duttonella* antigen type 1.2) [19], [28], [38], [39] in a relatively stable fashion. This VSG can be readily recognized by its specific 20 amino acid N-terminal sequence (ANNFAETDMEGVCTGALTLR) [30], [31]. As can be seen in Figure 1A, VSG-1.2 forward and VSG-1.2 reverse oligonucleotide primers were deduced from the full length *ILDat 1.2* gene sequence and flanking this 20 amino acid specific sequence. PCR reactions were then used to ascertain the identity of the initial IL 1392 *T. vivax* stabilate used in the present work, as previously shown [30], [31]. VSG-1.2F and VSG-1.2R primers amplified a 148 bp fragment of genomic DNA in IL 1392 bloodstream forms, and when sequenced, this showed more than 99% similarity with *ILDat 1.2* and only 2 point mutations as compared to Zaria Y486 (See Fig. 1B), confirming the presence of ILDat 1.2 VSG from the rodent infective West African *T. vivax* 1392 isolate, not reactive with Eastern *T. vivax* strains or with DNA from *T. brucei* and *T. congolense* [27], [28], [40] It is also worth noting that a PCR reaction comprising TvDNAF (forward) and TvDNAR (reverse) oligonucleotides amplified a 1,8 kb DNA product whose sequence was flanked by the two primers and presented 95% homology to the highly specie-specific ribosomal promoter of the Y486 *T. vivax* reference strain (not shown). Furthermore, we recently showed that the IL 1392 *T. vivax* genome possesses a functional proline racemase gene (*Tv*PRAC) that is absent in other trypanosomatid genomes [34]. Altogether these results established and confirmed the molecular identity of the IL 1392 *T. vivax* parasites used in the present work.

IL 1392 *T. vivax* bloodstream forms readily infected all mouse strains tested and were regularly maintained hereafter in the laboratory without losing infectivity through weekly passages in 7- to 8-week-old outbred Swiss (outbred) mice (CD-1, RJOrl:SWISS) (Janvier, France) by intra-peritoneal (i.p.) injection of 10^3^ parasite forms. As can be seen in Figures 1C and 1D, the parasites showed a predominantly slender morphology, an anterior free flagellum and a narrow posterior end containing a large sub terminal kinetoplast, similar to stained trypanosomes from cattle, as previously described [10], [23]. Figure 1E shows large numbers of *T. vivax* in blood, at the peak of parasitemia in outbred mice.

### Comparison of in vivo infections in mouse strains using *T. vivax* Y486-derived IL 1392 stock

Despite the high degree of gene synteny observed in kinetoplastids, genes coding for essential proteins associated with key metabolic reactions are not necessarily ubiquitous among members of the order. Accordingly, *T. vivax* but not *Trypanosoma brucei*, *Trypanosoma congolense* nor *Leishmania spp* possesses a *Tv*PRAC enzyme responsible for the interconversion of L- and D-proline enantiomers [34]. This enzyme, earlier described in *Trypanosoma cruzi* parasites, was shown to be essential for parasite metabolism and triggers non-specific polyclonal B cell responses in the host thus contributing to mechanisms of parasite escape from the host immune system [41], [42]. Taking into account the fact that *T. vivax* multiplies extracellularly in the host bloodstream, unlike the intracellular and extracellular *T. cruzi*, it is conceivable that *Tv*PRAC may also play a role in triggering non specific polyclonal B cell responses, contributing to antibody diversity, host immunosuppression, parasite evasion and persistence. In an attempt to further address these questions, we decided to develop a reliable and consistent mouse model and for this purpose studied several parasitological, hematological and immunological parameters of the infection using a parasite stock of defined antigenic identity.

The experimental murine infection was initially studied using 7 to 8-week-old intact BALB/c inbred mice infected with different inocula (10^1^ to 10^5^) of ILRAD 1392 *T. vivax* bloostream forms. The initial results showed that appearance of parasitemia was highly dependent upon the number of parasites injected as parasites could be detected as early as two days post-injection when a high inoculum (10^5^) was used, while three to six days were necessary when lower parasite numbers were injected (10^1^ to 10^4^) (not shown). Death seemed to correlate with parasite load since average time to death was also dependent on the number of parasites in the inoculum (not shown). Thus, the more elevated the parasite inoculum, the lower the survival rate, corroborating published data based on the original parasite isolate [43].

**Figure 2:**
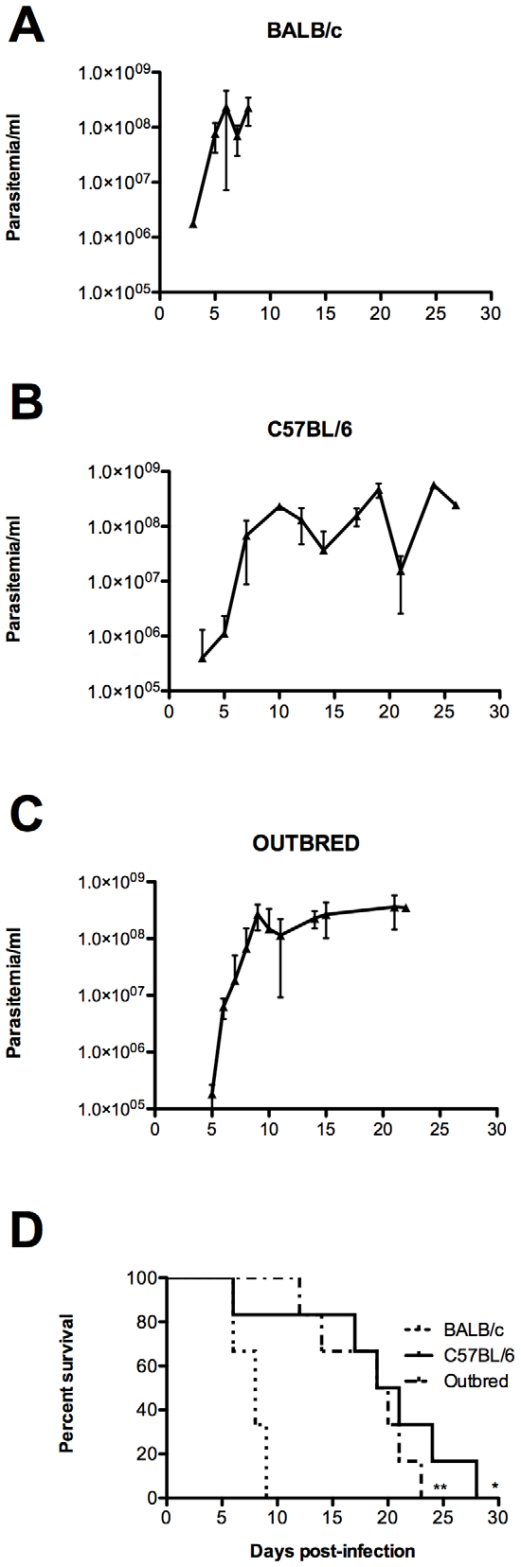
Effect of host background on parasite load and fate. BALB/c, C57BL/6 or Outbred mice were injected i.p. with 1×10^2^ bloodstream forms of *T. vivax* and the mean parasitemia recorded individually during infection (A, B, C). Mean mortality (D) is depicted compared to BALB/c mice. Results are given as arithmetic means ± standard deviations of at least three independent experiments. Cumulative mortality was recorded over time for all groups and Kaplan-Meir survival curves plotted for the three mouse strains (D); Comparison between survival curves was performed using Log-rank Mantel-Cox test: * p<0.028, ** p<0.0018, when compared with BALB/c survival.

In order to compare the impact of host genetic background on the establishment of infection, we then conducted studies using BALB/c (H2^d^), another inbred mouse strain (C57BL/6) which bears a different haplotype (H2^b^), and an outbred mouse stock, all infected with an inoculum consisting of 10^2^ bloodstream forms of *T. vivax*. BALB/c mice showed a rapid and pronounced increase in parasitemia that reached 4.10^8^ parasites/ml, as recorded by daily monitoring (Fig. 2A). As compared to BALB/c and C57BL/6, detectable parasitemia (4–6 days post infection - d.p.i.-, ≥10^4^ parasites/ml) was slightly delayed following infection of the outbred mice (Figures 2B and 2C). While parasitemia in all three mouse strains reached maximum levels 6 to 8 days post-infection, survival rates were significantly higher in the C57BL/6 and outbred mice than in the BALB/c mice which died in the first week of infection (Figure 2D). Moreover, while outbred mice showed a parasitemia plateau after 10 days of infection, recurrent parasitemia peaks were observed in the C57BL/6 mice over the same period, as also observed by De Gee et al. [7] and Mahan & Black [19], indicating that the C57BL/6 mice were partially controlling the parasite load.

### *T. vivax* experimental infection results in major changes in hematological parameters

**Figure 3:**
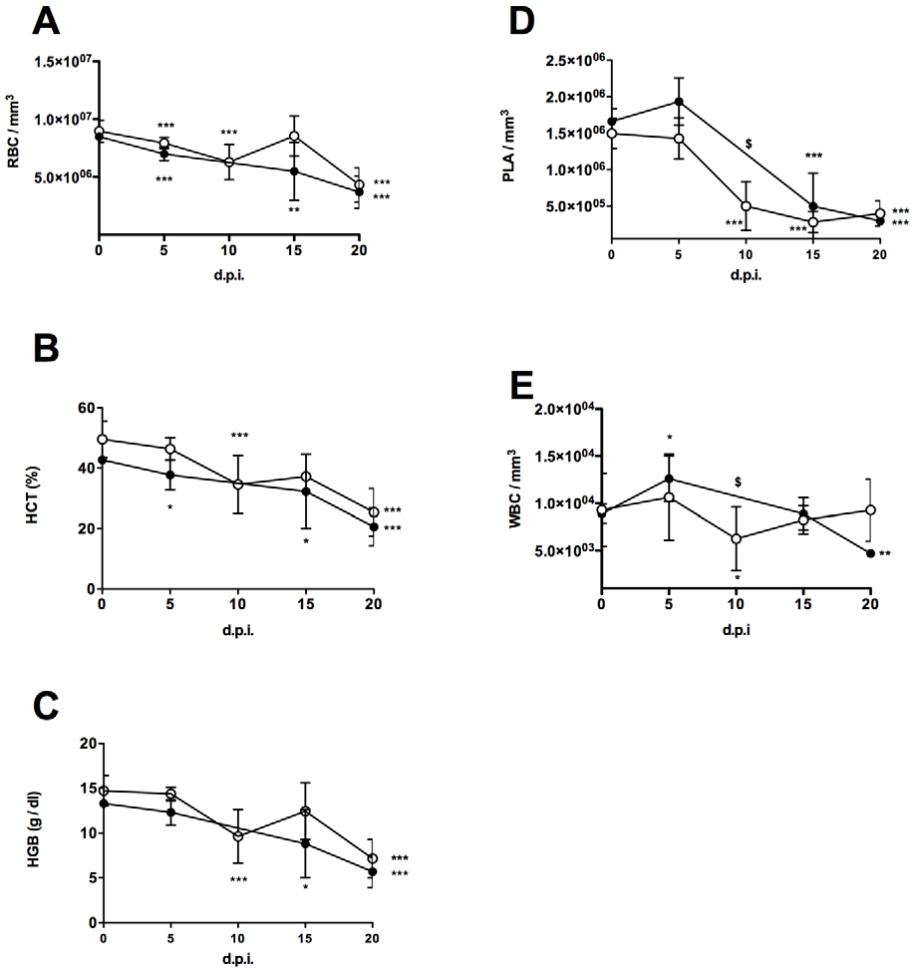
*T. vivax* induces major perturbations in hematological parameters and causes severe thrombocytopenia. 8-week-old Outbred (white symbols) or C57BL/6 (black symbols) mice were injected i.p. with 1×10^2^ bloodstream forms of *T. vivax*. Blood samples were collected individually every 2–3 days and red blood cell counts (RBC, A), hematocrit (HCT, B), hemoglobin concentrations (HGB, C), leukocytes (WBC, D) and platelets (PLA, E), were determined. Results are given for days 5, 10, 15 and 20 as arithmetic means ± standard deviations of at least three different experiments with 3–5 mice per time point/experimental group. *** p<0.001, ** p<0.01, * p<0.05, when compared with samples from day 0. $ = not determined on day 10 for C57BL/6 mice.

Since BALB/c mice proved to be highly susceptible to the infection, we continued our studies using only C57BL/6 and outbred mice as these were able to endure the infection over a longer period of time. Microscopic examination of the peripheral blood of infected animals indicated an apparent loss of red blood cells, concomitant with high levels of parasitemia. To monitor this phenomenon, peripheral blood samples taken from individual mice were analyzed throughout the infection and subjected to hematological analysis. Complete blood counts showed similar and severe changes in both mouse strains (Figure 3). Firstly, hemoglobin concentrations were significantly decreased in both C57BL/6 (from 13.3±0.1 to 5.7±1.0 g/dl) and outbred mice (14.7±0.2 to 7.2±0.6 g/dl). This decrease was associated with a fall in the red blood cell counts (from 8.5±0.1 to 3.7±0.8 10^6^ cells/mm^3^ and from 9.0±0.1 to 4.3±0.4 10^6^ cells/mm^3^ in the C57BL/6 and outbred mice, respectively) and in hematocrit values (from 42.7±0.5 to 20.6±3.6% and from 49.6±0.9 to 25.4±2.4% in the C57BL/6 and outbred mice, respectively) (Fig. 3A, 3B and 3C). Taken together, these alterations indicated that the infection gave rise to severe anemia as reported for natural cases of bovine trypanosomosis caused by *T. vivax* [6], [44]. An evaluation made to measure immature red cell production in the blood showed a transient 5-fold increase in the number of reticulocytes 14 days post-infection (data not shown). Although only 20% of the injected animals were still alive 20 days post-infection, these results suggest that, at the time of death, the mice were suffering from regenerative, normocytic and normochromic anemia.

Also, severe thrombocytopenia reported as an universal complication in the course of trypanosome infections [45] was observed in both the outbred and C57BL/6 mice, stemming from a dramatic fall in the platelet count as early as seven to ten days post-infection (Figure 3D). In addition, and except during the initial increase in white blood cell counts seen in the first days of the infection, a leucopenia was observed as the infection progressed and was more pronounced in the C57BL/6 mice (Figure 3E). The number of circulating lymphocytes fell significantly during the second week of infection, more precisely at around 20 d.p.i for the outbred mice and was accompanied by an increase in neutrophils and monocytes, as previously described with another Y-486 derived strain of *T. vivax* and albino mice [22] (see accompanying paper).

### Marked to severe liver and spleen lesions

The extent of the tissue damage caused by *T. vivax* infection was assessed by means of anatomic pathological and histopathological examinations. Here, we chose to use the highly reproducible outbred model as it gave lower inter-individual differences within the mouse groups and sustained and elevated parasitemia. Outbred mice were infected with 1×10^2^ parasites and a general anatomic pathological assessment of disturbances was conducted 20 days post-infection. At necropsy, gross lesions were observed only in the spleen and liver; the other organs were macroscopically normal. The spleens were uniformly enlarged, thereby characterizing marked splenomegaly, but did not show any congestion. They were firm and a little blood oozed from the cut surfaces. Randomly scattered white or red foci, ranging from 1 to 5 mm in diameter were observed on the capsules and the cut surfaces. Livers showed discrete, pale red or sometimes white foci that were sharply delineated from the adjacent parenchyma. These foci varied in size from 0.5 to 2 mm.

### Histopathological analyses showed an elevated *T. vivax* load in the tissues observed along with multifocal inflammatory infiltrates in different organs, extramedullary hematopoiesis in the liver and cerebral edema

**Figure 4:**
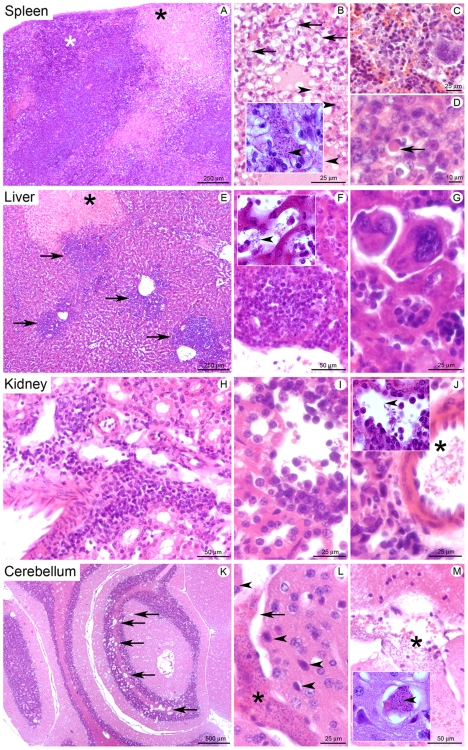
Histopathological study of mice infected with *Trypanosoma vivax*. 8-week-old Outbred mice were injected i.p. with 10^2^ bloodstream forms of *T. vivax* and different lymphoid and non lymphoid organs were harvested for histopathological examination 20 days post-infection. Spleen (A–D): (A) Diffuse lesions characterized by large necrotic foci in the red pulp (black star), associated with lymphoid tissue disorganization in the white pulp (white star). (B) Infiltration of a necrotic focus by activated macrophages (top of the Fig., arrows) and trypanosomes (arrowhead shows very small basophilic points in the inset depicting a higher magnification). (C) Presence of lower density hematopoiesis compared to non-infected mice. (D) Infiltration of the white pulp by activated macrophages and presence of a Mott cell (arrow). Liver (E–G): (E) Multifocal inflammatory lesions centered on portal tracts/centrilobular veins (arrows), and focal necrotic focus (star). (F) Peri-venous inflammatory infiltrate composed of plasma cells (mostly), but also lymphocytes and macrophages. In the inset depicting a higher magnification, arrowhead points to trypanosomes in the vascular spaces. (G) Foci of extramedullary hematopoiesis. Kidney (H–J): (H) Interstitial inflammatory infiltrates (I) mostly composed of plasma cells. (J) Trypanosomes in an arcuate artery (star); in the inset depicting a higher magnification, arrowhead points to trypanosomes in the vascular spaces. Cerebellum (K–M): (K) Multifocal lesions centered on blood vessels (arrows). (L) Blood vessel lumen filled by trypanosomes, proteins and erythrocytes (star), with perivascular edema (arrow) and ischemic neurons (arrowheads). (M) Trypanosomes in a meningeal blood vessel. Hematoxylin-eosin staining, scale bars are indicated at the bottom of each photograph. In the inset depicting a higher magnification, the arrowhead points to trypanosomes.

The tissues of outbred mice infected with *T. vivax* were also subjected to histopathological analysis 20 days post-infection. Lymphoid and non-lymphoid organs showed significant lesions (Figure 4). The spleens of infected animals (Figures 4A to 4D), showed diffuse lesions, more diffuse at the periphery of the organ, involving both the red and white pulps (Figure 4A). Lesions in the red pulp were characterized by necrosis with replacement of the normal tissue by an acidophilic and amorphous to fibrillar material containing cell debris, fibrin, extravasated erythrocytes and trypanosomes that were often clustered together in hemorrhagic foci (Figure 4B and inset arrowhead). Extramedullar hematopoiesis foci were reduced in number (Figure 4C). The white pulp showed disorganized lymphoid structure associated with marked infiltration by activated macrophages displaying vesiculous, euchromatic and nucleolated nuclei and abundant acidophilic cytoplasm. Infiltration by several lymphocytes and plasma cells was also noted (Figure 4D). Numerous plasma cells called ‘Mott cells’, characterized by their round shape and a polar cytoplasm containing stored immunoglobulins (Russel bodies), were observed in both the red and white pulps (Figure 4D, arrow). Collectively, these lesions were characteristic of diffuse, sub acute, necrotizing and hemorrhagic splenitis, associated with intralesional trypanosomes.

A multifocal to coalescing lesion, primarily centered on the portal tracts but also involving centrilobular veins, was observed in the liver (Figure 4E). As can be seen, the lesion was characterized by marked infiltration of plasma cells, lymphocytes and macrophages (Figure 4F). Trypanosomes were frequently observed in the vascular spaces, i.e. sinusoids and the portal and terminal hepatic veins. Many necrotic foci were also observed, randomly distributed in the liver parenchyma and associated with hemorrhages and trypanosomes (Figure 4F, inset arrowhead). A very high density of extramedullar hematopoiesis foci was noted in the liver sinusoids (Figure 4G). Collectively, these lesions were characteristic of multifocal to coalescing necrotizing and hemorrhagic hepatitis, associated with extramedullary hematopoiesis and intralesional trypanosomes.

The infection also induced bilateral, multifocal and sub acute tubulointerstitial nephritis, as can be seen in Figures 4H to 4J. The kidneys showed bilateral multifocal lesions, mostly involving the renal cortex and characterized by interstitial perivascular and periglomerular infiltration of plasma cells, lymphocytes and macrophages (Figures 4H and 4I). Very few neutrophils were seen in this lesion. Some randomly distributed tubular epithelial cells were noted, showing acidophilic cytoplasm and a condensed hyper basophilic (pycnotic) and/or fragmented nucleus (necrotic cells). Trypanosomes were observed in the blood vessels, mostly in the arcuate arteries at the corticomedullary junction (Figure 4J, star and 4J, inset arrowhead).

The histopathological investigation of the central nervous system also discovered multifocal lesions centered primarily on small and medium-sized veins, and more severe in the cerebellum (Figure 4K). Evidence was often noted of dilatation and filling of the blood vessel lumen by erythrocytes, proteins and numerous trypanosomes. Vasogenic edema was observed, characterized by the presence of an amorphous and unstained material accumulated in perivascular spaces. Some angular and shrunken neurons close to these lesional blood vessels contained acidophilic cytoplasm and a condensed hyper basophilic nucleus, characteristic of ischemic necrosis (Figure 4L). Trypanosomes were also seen in the meningeal blood vessels (Figure 4M, star and inset, arrowhead).

Interestingly, and although observed in only a few animals, moderate lymphoid hyperplasia was noted in the lymph nodes, apparently associated with intravascular or intrasinusal trypanosomes, as previously described in ruminants [46] (not shown). In addition, histopathological examination of the heart, revealed the presence of numerous parasites in the ventricular cavities as well as in the blood vessels located in the periphery of the myocardium in some mice. Some of these lesions were accompanied by an infiltration of mononuclear cells (multifocal myocarditis), i.e. plasmocytes, lymphocytes and macrophages. These findings are suggestive of a myocardial commitment induced by the infection and are consistent with the congestive heart failure that has previously been reported for cattle trypanosomosis [26], [47], [48].

## Discussion

While Human African Trypanosomiasis (HAT) has drawn the attention of many research groups over the last three decades, lesser consideration has been given to animal trypanosomosis (*Nagana*) despite its considerable impact on the development and fertility of livestock and the economical hardship it causes in several countries. Most studies, both in the distant past and more recently, have concentrated on analyzing the genetic factors involved in tolerance to trypanosomosis, or on describing the general deregulation of the immune response as expressed by a few individuals in different cattle species in the field [13], [49], [50], [51], [52], [53], [54], [55], [56]. For instance, *T. brucei,* which is of little clinical importance in livestock, has generally constituted the parasite of choice in experimentally and genetically controlled studies [57], [58], [59], [60]. But trypanosomosis, which is overwhelmingly the most prevalent cattle illness in Africa and South America, is mainly caused by *T. vivax* and *T. congolense*. The hallmark of their pathogenesis in the field is severe anemia accompanied by a general immunosuppressive condition [6], [21]. Since various types of tissue damage have been described for ruminants, horses, sheep and goats, distinct strategies are used to explain host resistance (“tolerance”?) or susceptibility to trypanosomes and/or the etiology of the lesions and tissue damage observed [20], [25], [61], [62], [63]. To overcome these difficulties, several experimental models were developed in rats and mice (see [21] for a review). Athough these studies showed that the mouse was potentially an important tool in understanding the pathogenesis of trypanosomosis and most particularly the immunobiology of host-parasite interactions, most subsequent studies focused on trypanosomes of the subgenus *Trypanozoon* (i.e. *T. brucei*) leaving *T. (Duttonella) vivax* infections poorly characterized. Thus, despite the fact that remarkable progress was made, the array of features shown by diverse stocks of *T. vivax* isolates has not painted a clear picture of the factors that could be central to the development of appropriate immuno(chemo)therapies [15], [64].

To better investigate the relationship between trypanosomosis and genetically-controlled rodents, we therefore undertook to develop new mouse models of *Trypanosoma (Dutonnella) vivax* infection. This parasite not only differs from other trypanosomes belonging to the *Trypanozoon* subgenus (i.e. *T. brucei* and *T. equiperdum, but not T. evansi*) with regard to its transmission and tissue distribution in the host, but also is generally recognized as possessing diverse isolates which may or not express the ability to infect laboratory rodents [10], [23], [36],[65]. For instance, East African isolates are known to induce mild infections and hemorhagic syndromes in cattle and only some stocks are adapted to rodents. Conversely, West African *T. vivax* isolates, obtained at different stages of natural infection, are responsible for the majority of trypanosomosis cases in cattle and other ruminants and may express mild, intermediate or high virulence to mice [7], [65]. The work presented here used a well characterized West African *T. vivax* isolate which is infective to rodents (IL 1392) [19], [23], [28], [31], [38], [39] and describes in detail the parasitological, hematological and histopathological parameters of the infection in different inbred and outbred mouse strains. IL 1392 was chosen for its stable expression of VSG ILDat 1.2, characteristic of rodent-adapted West African *T. vivax* isolates that together with closely related South American stocks pertain phylogenetically to the same clade [66]. Initially, our studies showed that the IL 1392 isolate, retained its infective characteristics and mouse infection profile after a long cryopreservation period (see Material and Methods) [11], [22]. In addition, the experimental mouse models used in this work proved easy to handle on infection and reflected the general characteristic features observed in livestock, namely the remodeling of secondary lymphoid organs, cardinal severe anemia, genetically-related differences in resistance to the parasite (but not to death) and the development of multifocal tissue hemorrhages, necrosis and consequent systemic pathologies.

Briefly, the work confirmed widespread observations that BALB/c mice are highly susceptible to *T. vivax* infection with the shortest survival time as compared to C57BL/6 or outbred mice. Furthermore, the BALB/c but also C57BL/6 and outbred experimental mouse models showed an early exponential increase in parasitemia, closely resembling acute trypanosomosis in the field [46]. Regardless of the fact that C57BL/6 and outbred mice proved to be more “tolerant” than BALB/c to *T. vivax,* limiting the pathological consequences of the infection and delaying mortality, all the mice presented common pathognomonic signs of the disease, such as anemia, cachexia, thrombocytopenia associated with high parasitemia and a tendency to leucopenia in the terminal stages [22], [45], [67]. As frequently observed in cattle and goats infected by *T. vivax*, the histopathological analysis of mouse tissues committed by the infection showed accumulation of trypanosomes nuclei and debris in the blood vessels of mouse spleen, liver and brain. Some extravascular foci associated with intense inflammatory and degenerative tissue disorders and cerebral edema were also observed [5], [22], [68]. It is noteworthy that both the red and white pulps of the spleen showed severe necrosis, with germinal centers depleted of lymphocytes, which could explain the lymphopenia observed at late stages in the infection. The marked splenitis, hepatitis and central nervous system involvement with vasogenic oedema and ischemia, reflect to what extent the model is of value in studies of *T. vivax* pathogenesis. The characteristic erythrocytopenia experienced at the peripheral level is suggestive of a decreased erythropoiesis. However, we cannot rule out possible extravascular haemolysis, or more specific apoptosis due to an autoimmune process triggered by the infection. A detailed study of bone-marrow cell populations before and during the infection will better address these questions (see accompanying paper).

The present systematic analysis described here using different mouse strains infected with a well characterized West African *T. vivax* isolate showed that these models are reliable and constitute new experimental tools for the study of trypanosomosis. The studies conducted aimed to develop models that could be used in the future to gain further insight into the genetic mechanisms involved in drug resistance and the discovery of new drug targets for purposes of parasite control. However, further studies using Eastern or other Western *T. vivax* stocks and the present mouse strains are encouraged to better approach the influence of genetic parasite divergences on disease outcome. These models of infection will be useful in the *in vivo* testing of new chemicals against *T. vivax* trypanosomosis since tests in the definitive hosts are prohibitively expensive. Finally, the mouse models given herein provide a description of the parasitological, hematological and histopathological features of *T. vivax* infection and pave the way to a more in-depth understanding of the immune responses involved in disease tolerance and susceptibility to *T. vivax*.
